# Aluminum-target-assisted femtosecond-laser-filament-induced water condensation and snow formation in a cloud chamber

**DOI:** 10.1038/s41598-018-36548-0

**Published:** 2018-12-27

**Authors:** Yonghong Liu, Jiansheng Liu, Haiyi Sun, Jingjing Ju, Xinkai Hu, Cheng Wang, Yuxin Leng

**Affiliations:** 1 State Key Laboratory of High Field Laser Physics, Shanghai Institute of Optics and Fine Mechanics, Chinese Academy of Sciences, No. 390, Qinghe Road, Jiading District, Shanghai 201800 China; 2 Department of Physics, Shanghai Normal University, Shanghai, 200234 China; 3 Institute of Modern Optics, Nankai University, Tianjing, 300000 China; 4 IFSA Collaborative Innovation Center, Shanghai Jiao Tong University, Shanghai, 200240 China

## Abstract

We compare the water condensation and snow formation induced by a femtosecond laser filament with that when the filament is assisted by an aluminum target located at different positions along the filament. We reveal that the laser-filament-induced water condensation and snow formation assisted by the aluminum target are more efficient compared with those obtained without the assistance of the aluminum target. We find that the mass of the snow induced by the laser filament is the largest when the aluminum target is located at the end of the filament, smaller when it is at the middle of the filament, and the smallest at the beginning of the filament. These findings indicate that a higher plasma density and the generation of vortex pairs below the filament are important for enhancing the efficiency and yield of the laser-induced water condensation and precipitation. The higher plasma density provides more cloud condensation nuclei and facilitates the water condensation; vortex pairs below the filament are favourable to the growth of particles up to larger sizes.

## Introduction

Femtosecond laser filamentation generated by high-power ultrashort laser pulses originates from the dynamic balance between Kerr self-focusing and defocusing by the self-generated plasma and/or higher-order Kerr terms^1,2^. The resulting filament can deliver an intensity as high as 5 × 10^13^ W· cm^−2^, sufficient for ionizing and photo-oxidizing air^3,4^, and it can induce airflow motion^5,6^. Laser filaments have attracted significant research interests owing to their potential for atmospheric applications^7^ such as remote sensing^8–10^, the triggering of high-voltage discharges^11,12^, and air lasing^13–15^ as well as laser-induced condensation^16–22^. Recently, it has been demonstrated that a femtosecond laser filament can induce water condensation in a cloud chamber and the atmosphere^17–19^. Several studies on this subject have been reported^20–28^. The femtosecond laser filament can locally generate large ozone and NO_x_ concentrations in the air through the multiphoton dissociation and ionization of oxygen, which leads to the production of HNO_3_, trace gases, and oxidized volatile organics^26^. The hygroscopic HNO_3_ is identified as the major contributor to the particle growth in the form of NH_4_NO_3_ for a relative humidity larger than 70%. It has been demonstrated that trace gases and oxidized volatile organics enhance the laser-induced nucleation of new particles and increase the concentration of laser-induced particles^26^. Using different laser pulses and ambient gases in laser-induced water condensation^22,23,27,28^, it has been revealed that ultraviolet (UV) ultrashort pulses can provide a significantly higher efficiency for particle generation compared with that obtained using near-infrared (NIR) pulses^22,23^. Further, it has been shown that the mass of snow induced by laser filaments in argon is larger than that in air or helium^27^. In addition, it has been suggested that the laser-induced airflow has a significant effect on water condensation and precipitation^5^. The airflow can create a supersaturated condition, under which it is easy for the existing small particles to grow into large particles^5,25^.

Laser filaments can be created remotely and can last for tens or even hundreds of meters in the atmosphere. In addition, it has been demonstrated that they can propagate through clouds and turbulence^3,4,29^. Therefore, they are promising candidates for weather modification applications^7^. However, as an outdoor-orientated technique, one of the most concerning questions is how to increase the yield and efficiency of laser-induced water condensation. In this study, we investigated the enhancement of plasma density effect on the water condensation and snow formation induced by femtosecond laser. Since the metallic target can provide a higher plasma density under laser filament irradiation, an aluminum target available material in our lab was chose. It was located at different positions along the filament in the experiments. The airflow and snow yields were compared for two cases of a laser filament only and a laser filament including the presence of the aluminum target located at different positions along the filament. We observed that aluminum-target-assisted femtosecond-laser-filament-induced water condensation and snow formation were more efficient than those obtained using the laser filament only. The mechanisms of these phenomena were discussed on the basis of the interaction between the femtosecond laser filament and the metallic target.

## Experimental Setup

The experimental setup is very similar to those reported in our previous studies^27,28^. The experiments were conducted using a Ti: sapphire femtosecond laser system that delivered laser pulses of up to 9.5 mJ per pulse with a spot diameter of 12 mm, which had a pulse duration of 30 fs (a peak power of 317 GW), a wavelength of 800 nm, and a repetition rate of 1 kHz. The laser pulses were focused using a lens with a focal length of *f* = 300 mm and generated ~2.8-cm long filaments in a cloud chamber (its length, width, and height were 50, 50, and 20 cm, respectively). According to the images of the beam spot recorded on a white screen at a distance of 2 meters from the filaments, multiple filaments were formed. An aluminum target was located at different positions along the filament to interact with the laser filament. A 532-nm continuous-wave (CW) laser beam was used as a probe beam. The probe beam was enlarged and truncated using a slit with a height and width of 40 mm and 5 mm, respectively. Then it copropagated with the femtosecond laser beam into the chamber at normal incidence with respect to the surface of the aluminum target. The external surfaces of the chamber were covered with a 2.5-cm-thick insulated foam to maintain a stable temperature in the chamber. The vertical temperature gradient and relative humidity inside the chamber were adjusted using a refrigerating machine to cool the bottom of the chamber and an electric current of a heating wire submerged in the water reservoir, respectively. During the experiments, the temperature of the cold bottom base plate of the chamber was set to −50 °C, while the top plate of the chamber was maintained at room temperature. The power of the heating wire was set to be 19.2 W. A water reservoir filled with distilled water was mounted at a height of 17 cm relative to the cold bottom base plate. The images of the filaments and the Mie scattering of the probe laser around the filament were recorded from a side window of the chamber using a digital camera (Nikon D7000). The spectral signals of the interaction between the filament and the aluminum target were detected from another window on the other side of the chamber using a grating spectrometer (Shamrock 303i, Andor).

## Results

**Figure 1:**
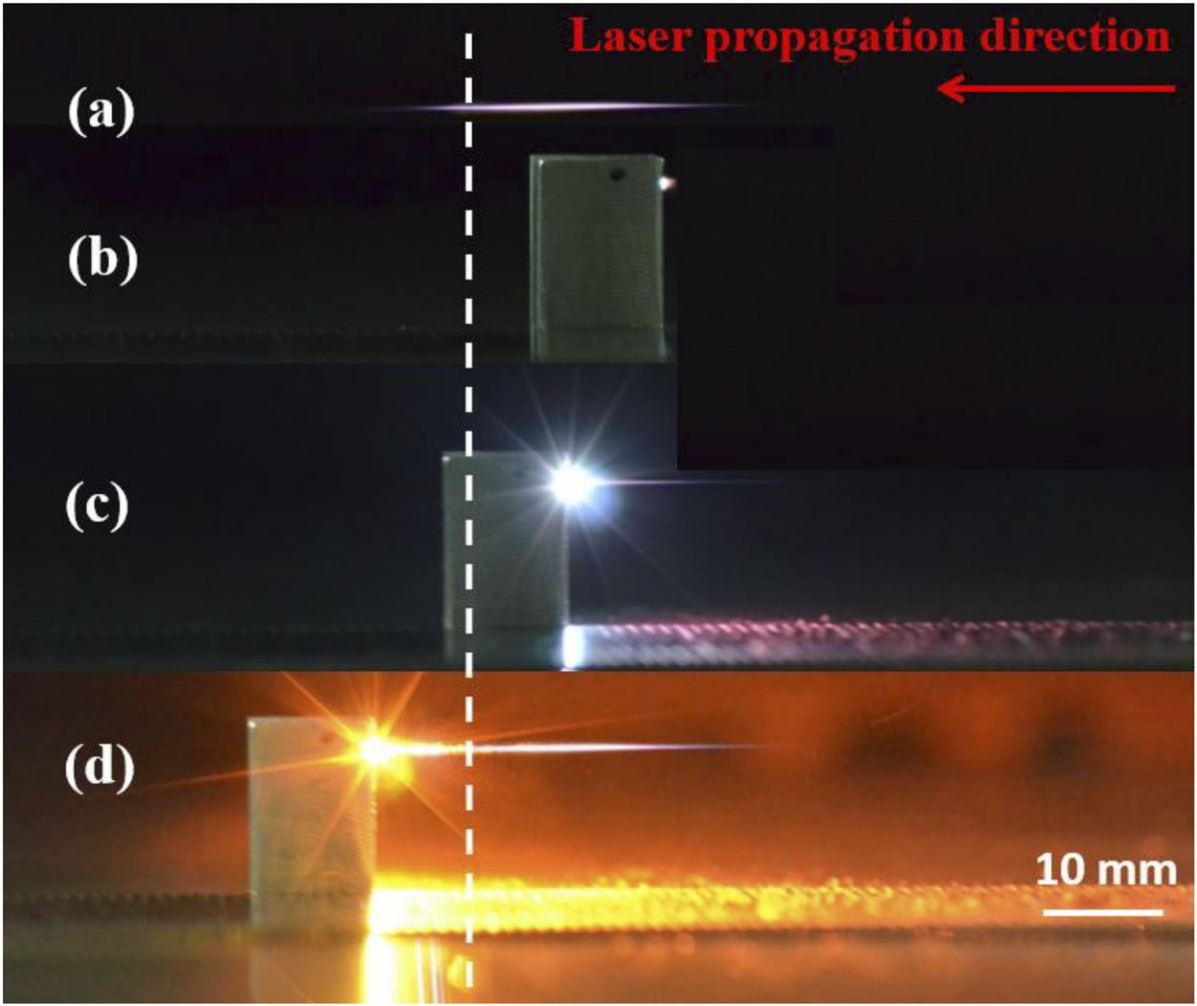
Side images of filaments (**a**) without the aluminum target and with the aluminum target located at the (**b**) beginning, (**c**) middle, and (**d**) end of the filament, recorded using a digital camera (Nikon D7000: *f* number (*F*) = 5.6, light sensitivity (ISO) = 800,shutter speed (*S*) = 1/13 s). The dashed line outlines the position of the geometric focus. The arrow indicates the propagation direction of the laser pulses.

**Figure 2:**
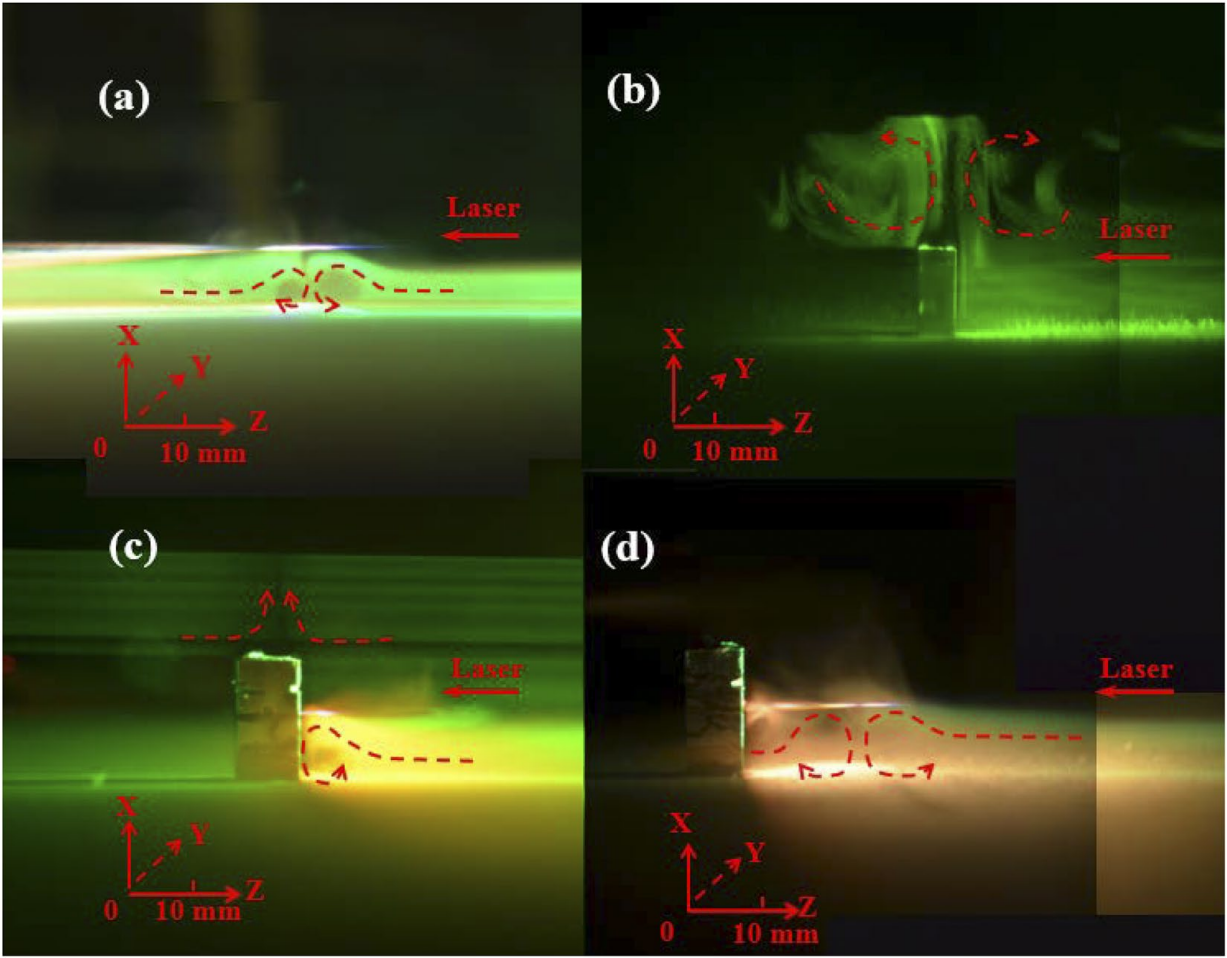
Side Mie scattering images (**a**) without the aluminum target and with the aluminum target located at the (**b**) beginning, (**c**) middle, and (**d**) end of the filament, recorded using a digital camera (Nikon D7000: *F* = 5.6, ISO = 800, *S* = 1/13 s). The dotted curves outline the rotation directions of the vortices.

**Figure 3:**
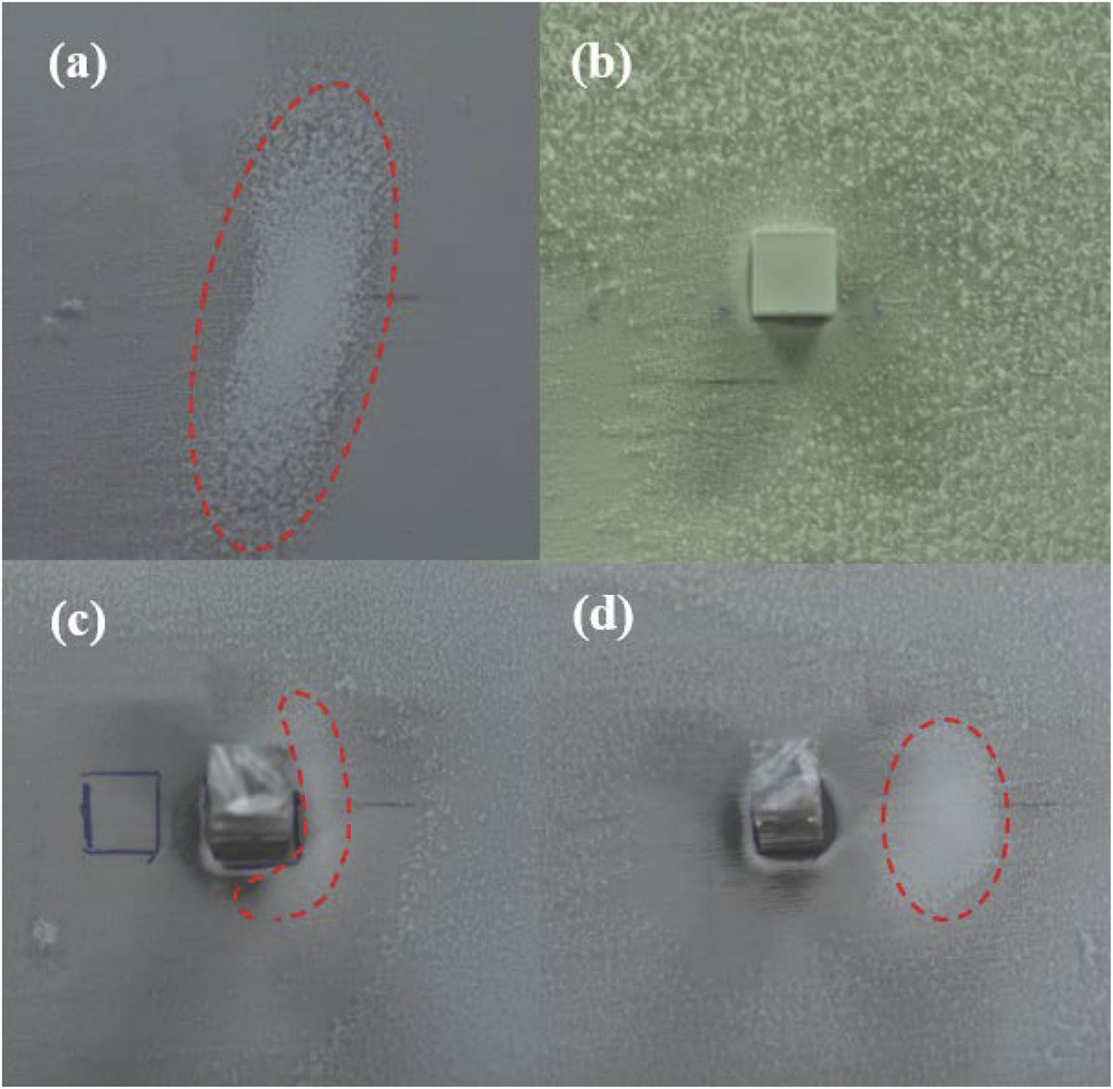
Recorded images of snow piles (**a**) without the aluminum target and with the aluminum target located at the (**b**) beginning, (**c**) middle, and (**d**) end of the filament after 40 min of irradiation by the femtosecond laser (Nikon D7000 digital camera: *F* = 5, ISO = 640, *S = *1/15 s for (**a**,**c**,**d**), and *F* = 5.6, ISO = 2500, *S = *1/30 s for (**b**)). The dashed lines outline the heaps of snow formed below the filament center.

First, we investigated femtosecond-laser-filament-induced water condensation and snow formation using only the laser filament, the height of the laser axis relative to the bottom base plate of the cloud chamber was set at 1 cm. Then, we performed experiments with an aluminum target placed in the filament. Once the bottom base of the chamber was cooled for 20 min, the femtosecond laser pulses were focused into the chamber to generate femtosecond laser filaments, where the temperature and relative humidity were approximately −11 °C and 75%, respectively. Figure 1(a–d) show the recorded images of the filaments from the side of the chamber using only the laser filament and placing the aluminum target at the beginning, middle, and end of the filament, respectively. These Figs show that the interaction of the filament with the surface of the aluminum target produces a bright spark and plasma ejection that depends on the position of the aluminum target along the filament. It is noted that the intense interaction was visible at the both the middle and end of the laser filament. Airflows around the filament and aluminum target were observed, as shown in Fig. 2(a–d). The vortex pairs that have opposite rotation directions were observed below the filament center when the aluminum target was located at the end of the filament [Fig. 2(d)]. This is similar to the case without the aluminum target [Fig. 2(a)] and consistent with our previous experimental results^5,6,19,27,28^. Owing to the obstruction of the aluminum target, the filament was not observed when the aluminum target was located at the beginning of the filament. Only a single counter-clockwise vortex appeared below the filament center when the aluminum target was located at the middle of the filament, as shown in Fig. 2(c). Similar vortex pairs that have opposite rotation directions were visible above the aluminum target center when the aluminum target was located at the beginning and end of the filament. After 40 min of irradiation by femtosecond laser pulses, a layer and heap of snow were formed on the cold bottom plate and below the filament center, respectively, as shown in Fig. 3(a–d). In Fig. 3(b), although there is no visible snow heap below the filament center, a layer of snow was observed on the cold bottom plate. The size of the snow particles that cover the entire cold plate [Fig. 3(b)] is larger, and the snow is looser compared with the other three cases [Fig. 3(a,c,d)]. Using the same conditions (60 min cooling, 40 min laser irradiation), we collected the snow that covered the entire cold plate in the four different cases and the background snow. The net amount of snow was obtained by subtracting the amount of background snow. Then, the water obtained from the melted snow was analyzed respectively using an ion chromatograph (Dionex500) and an Inductively Coupled Plasma (ICP) emission spectrometer (Leeman Prodigy). Table 1 summarizes the net snow masses and the ${{\rm{NO}}}_{3}^{-}$, ${{\rm{SO}}}_{4}^{2-}$and Al^3+^ concentrations for the different cases, averaged over three measurements. The results in Table 1 indicate that the concentration of HNO_3_ is the highest without the assistance of the aluminum target, while the mass of snow is the largest when the aluminum target is located at the end of the filament (twice the mass obtained without the assistance of the aluminum target). Lower concentrations of ${{\rm{SO}}}_{4}^{2-}$ were detected in each case. The concentration of Al^3+^ [Table 1(c)] is higher compared with the other two cases [Table 1(b,d)] due to larger diameter at the middle of the filament. In addition, we measured the mass of the aluminum target before and after the experiment. It is found that the reduced masses of the aluminum target are 0.3, 0.5, and 0.2 mg when it is located at the beginning, middle, and end of the filament, respectively. The reduced mass of the aluminum target is almost stable under the same experimental conditions.

**Table 1:** Net snow masses on the entire bottom cold plate and ${{\rm{NO}}}_{3}^{-}$, ${{\rm{SO}}}_{4}^{2-}$ and Al^3+^ concentrations in the water obtained by the melted snow for the different cases: (a) without the aluminum target and with the aluminum target located at the (b) beginning, (c) middle, and (d) end of the filament.

Cases Unit	Snow mass (g)	${{\bf{NO}}}_{{\bf{3}}}^{{\boldsymbol{-}}}$ concentration (ppm)	${\bf{s}}{{\bf{o}}}_{{\bf{4}}}^{{\bf{2}}{\boldsymbol{-}}}$ concentration (ppm)	Al^3+^ concentration (ppm)
(a)	1.8977 ± 0.1960	86.23 ± 2.67	0.76 ± 0.03	
(b)	2.9344 ± 0.2776	38.36 ± 0.66	0.21 ± 0.01	21.58 ± 0.32
(c)	3.3271 ± 0.3954	42.23 ± 1.45	0.29 ± 0.04	24.39 ± 0.62
(d)	3.6910 ± 0.4818	64.00 ± 1.83	0.56 ± 0.06	18.44 ± 0.15

The errors were obtained by the standard deviations.

## Discussion

**Figure 4:**
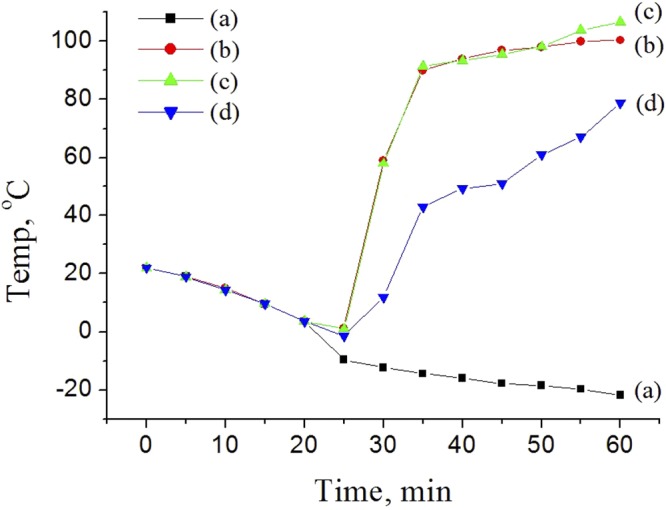
Dependence of the temperature of the aluminum target on the time (**a**) without laser irradiation during the experiment and when the aluminum target is located at the (**b**) beginning, (**c**) middle, and (**d**) end of the filament.

In order to explain the differences between the masses of the femtosecond-laser-filament-induced snow with the assistance of the metallic target located at different positions of the filament, we analyze the interaction between the laser pulses and the aluminum target. During the interaction process, laser energy is first absorbed by the solid surface through multiphoton, tunnel, and impact ionizations processes^30^. Subsequently, hot-electron relaxation^31–33^, shock waves^32,34,35^, ultrafast melting and vaporization^30^, plasma ejection^35,36^, and subsequent resolidification occur through the ablation process^30^. In our experiment, a large amount of plasma is ejected when the femtosecond laser filament interacts with the aluminum target. Owing to the lower ionization potential of the aluminum target, a high-density plasma is created by the irradiation of the intense laser filament. In addition, irreversible material breakdown and ablation occur. Owing to the collisions between electrons and ions, part of the absorbed energy is transferred to the ions and lattice. Therefore, part of the laser energy is transferred into the aluminum target. Consequently, the aluminum target is heated by the femtosecond laser filament. The heating drives the increase in the hydrodynamic pressure, which causes a violent ejection of hot matter away from the aluminum target and the generation of shock waves. The heated aluminum target is a heat source that can induce a disturbance in the airflow and the generation of vortex pairs above the aluminum target, as shown in Fig. 2(b,c). To clarify this deduction further, we measured the temperature of the aluminum target. In the experiment, a temperature detector was tied to the back of the aluminum target, and the front surface of the aluminum target interacted with the different positions of the filament: the beginning, middle, and end of the filament. Figure 4 shows the results of experimental measurements of the temperature of the aluminum target during laser filament irradiation. Owing to the clamped intensity of the filament, the dependence of the temperature of the aluminum target on the heating time is similar when the aluminum target is located at the beginning and middle of the filament. The temperature gradually increases and then it reaches a plateau at ~100 °C. When the aluminum target is located at the end of the filament, the temperature is lower owing to the decreased intensity at the end of the filament; in this case, vortex pairs were not clearly observed above the target [Fig. 2(d)]. On the other hand, vortex pairs were observed in the other two cases [Fig. 2(b–c)] owing to the large sizes of the particles^6^. However, the amount of snow is the largest when the aluminum target is located at the end of the filament (Table 1). This indicates that the formation of vortex pairs above the aluminum target is not the main factor for the increase in the snow yield. The weak vortex pair above the aluminum target is most likely caused by the smaller temperature gradient above the aluminum target in our cloud chamber. This is not conducive to the formation of a supersaturated condition^25^.

**Figure 5:**
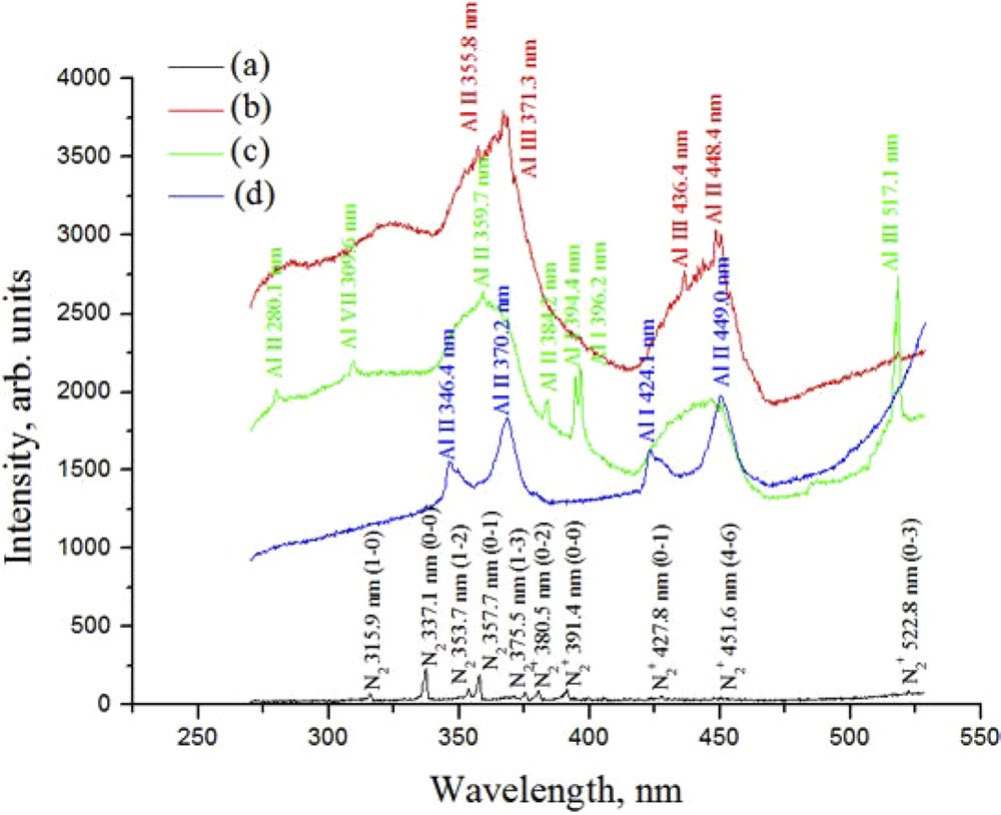
Measured side-spectra lines: (**a**) with the laser filament only and when the aluminum target is located at the (**b**) beginning, (**c**) middle, and (**d**) end of the filament. The slit width and exposure time for collection using a grating spectrometer with a grating with 300 grooves/mm are: (**a**) 100 µm and 500 ms, (**b**) 100 µm and 10 ms, (**c**) 50 µm and 8.24 ms, and (**d**) 100 µm and 1000 ms, respectively. The nitrogen lines were identified using the results reported in ref.^39^.

The intense femtosecond laser filament can produce electrons, ions, and other particles in air. These particles can synthesize condensable species such as volatile organic compounds and sulfuric- and nitric-acid compounds by photolysis and photo-oxidation reactions. They can initiate water condensation and are often considered as an important source of cloud condensation nuclei (CCN). In addition, in our experiment, the interaction of the laser filament with the aluminum target generated numerous charged particles. These particles contained ejected material with different sizes, aluminum atoms, ions, etc. In addition, the ablated trace aluminum would from nanoparticles. All of these particles could be an important source of CCN in the water condensation process. In order to verify that the particles emerged from the irradiation between the laser filament and the aluminum target, we measured the side spectra of the filament, as shown in Fig. 5. Aluminum spectral lines were observed^37^, which demonstrate the generation of a large number of aluminum ions and atoms. A higher plasma density assisted by the aluminum target increases the efficiency of snow formation; this is confirmed by the experimental results, which show that the amount of snow was significantly larger when the laser filament was assisted by the aluminum target compared with that without the aluminum target.

It is found in Fig. 2 and Table 1 that another important factor affecting the amount of snow is the formation of a vortex pair below the filament, where a supersaturation condition emerges owing to the large temperature gradient below the filament^25^. Therefore, in the following, we analyze the difference in the amount of snow by considering both the vortex pair below the filament and the plasma density. When the aluminum target is located at the beginning of the filament, the presence of intense vortex pairs below the filament is not likely owing to the absence of a long filament. However, the plasma density should be higher than that without the assistance of the aluminum target owing to the intense avalanche ionization in the aluminum target. When the aluminum target is located at the middle of the filament, half of the laser filament induces a vortex below the filament center with a counter-clockwise rotation. Considering the clamped intensity along the filament, the plasma density should be higher than that at the beginning of the filament. When the aluminum target is located at the end of the filament, a plasma with a high density and vortex pairs below the filament emerge, as the intensity at the end of the filament is sufficiently higher than the ionization potential of the aluminum target, similar to that at the beginning and middle of the filament, as demonstrated by the measurement results in Figs 4 and 5. In addition, the plasma density also includes the plasma induced by the overall filament in air.

A higher plasma density can provide more CCN to make particles easier to condense, and it would also deposit more heat into the air by plasma recombination. A larger heat release would cause stronger turbulence, where a continuous supersaturation environment is created around the filament. The shock waves that emerge from the interaction between the laser filament and the aluminum target also contribute to the formation of a supersaturation condition^38^. The supersaturation condition could allow efficient condensation; in addition, it could favor the growth of the particles. The results in Table 1 show that the mass of the collected snow is the largest when the aluminum target is located at the end of the filament. It decreases as the position of the aluminum target shifts towards the beginning of the filament and has the lowest value when the aluminum target is not included.

## Conclusion

We experimentally compared the airflow and the amount of water condensation induced by a femtosecond laser filament with those obtained when the filament was assisted by an aluminum target located at different positions along the filament in a cloud chamber. The femtosecond-laser-filament-induced water condensation and snow formation assisted with the aluminum target had significantly higher efficiencies compared with those when only the laser filament was employed. The amount of snow was the largest when the aluminum target was located at the end of the filament, where a higher plasma density and intense vortex pairs below the filament center emerged, which benefited water condensation and the growth of particles. These findings could contribute to real applications of laser-induced water condensation in the atmosphere.
